# Investigating the genetic and environmental aetiologies of non-suicidal and suicidal self-harm: a twin study

**DOI:** 10.1017/S0033291721000040

**Published:** 2022-11

**Authors:** Kai Xiang Lim, Georgina Krebs, Kaili Rimfeld, Jean-Baptiste Pingault, Frühling Vesta Rijsdijk

**Affiliations:** 1 Social, Genetic and Developmental Psychiatry Centre, Institute of Psychiatry, Psychology and Neuroscience, King's College London, London, UK; 2 National and Specialist OCD and Related Disorders Clinic for Young People, South London and Maudsley NHS Foundation Trust, London, UK; 3 Division of Psychology and Language Sciences, University College London, London, UK

**Keywords:** Self-harm, genetics, twin design, non-suicidal self-harm, suicidal self-harm, suicide attempts

## Abstract

**Background:**

Self-harm is a major health concern, not only as a signal of distress but also as a strong predictor of later suicide. Self-harm can be further refined into suicidal self-harm (SSH, i.e. suicide attempt) and non-suicidal self-harm (NSSH). Understanding the aetiologies of NSSH and SSH can help inform suicide prevention strategies. Using a twin design, we investigated the phenotypic and aetiological relationships between NSSH and SSH, and their aetiological overlap with mental health problems.

**Methods:**

We analysed data from the Twins Early Development Study using structural equation modelling. At age 21 years, 9063 twins (62.4% female) answered questions related to self-harm. At age 16 years, 19 self- or parent-reported mental health measures were administered, including measures of internalising and externalising problems, psychotic-like experiences and substance abuse.

**Results:**

Prevalences for NSSH and SSH were 21.9% and 10.5%, respectively. Additive genetic factors explained half of the variance in NSSH (55%) and SSH (50%), with the rest explained by non-shared environmental factors. Phenotypically, NSSH and SSH were strongly correlated (*r* = 0.87) with their correlation explained by genetic (57%) and non-shared environmental (43%) factors. We found no evidence that NSSH and SSH differed in their phenotypic and aetiological relationships with mental health measures.

**Conclusion:**

Our findings suggest no aetiological difference between NSSH and SSH. NSSH and SSH should be regarded as two different ends of a continuum, rather than as two distinct categories.

## Introduction

Self-harm can be defined as any act of self-injury and self-poisoning carried out by an individual, regardless of intention or motivation (Hawton et al., 2003). It is a major public health concern. Not only distressing and impairing, it is also predictive of suicide (Hawton, Zahl, & Weatherall, 2003b), which is the second leading cause of death in young adults (World Health Organization, 2014).

Self-harm can be separated into non-suicidal self-harm (NSSH) and suicidal self-harm (SSH), i.e. a self-harm episode without suicidal intention, or suicide attempt. In the fifth edition of the Diagnostic and Statistical Manual of Mental Disorders (DSM-5), self-harm was suggested to be divided along the dimension of suicidal intention as two separate conditions, i.e. non-suicidal self-injury (NSSI) and suicidal behaviour disorder (American Psychiatric Association, 2013).

There is no consensus yet whether NSSH and SSH should be viewed as two distinct entities (Butler & Malone, 2013; Kapur, Cooper, O'Connor, & Hawton, 2013). At the phenotypic level, NSSH and SSH co-occur frequently (Klonsky, May, & Glenn, 2013; Nock, Joiner, Gordon, Lloyd-Richardson, & Prinstein, 2006). Studies have also shown that SSH is associated with higher levels of psychological and psychosocial impairment than NSSH (Bjureberg et al., 2019; Jacobson, Muehlenkamp, Miller, & Turner, 2008). Hence, it was proposed that self-harm should be regarded as opposite ends of the same continuum of self-harm behaviours (Brausch & Gutierrez, 2010; Linehan, 1986; Stanley, Winchel, Molcho, Simeon, & Stanley, 1992), with NSSH considered as a gateway towards more severe form of self-harm (i.e. SSH). Another theory, the interpersonal theory of suicide, proposes that NSSH increases one's acquired capability for suicide through repeated exposure to painful and/or fearful experiences, and when this is joined with perceived burdensomeness and thwarted belongingness, it will lead towards lethal SSH (Joiner, 2007; Van Orden et al., 2010).

Nonetheless, these two facets of self-harm are also different in terms of prevalence, frequency, lethality of methods, and attitudes towards life and death (Muehlenkamp & Gutierrez, 2007). Indeed, a recent study in an UK adolescent sample suggests that although NSSH and SSH may share some common risk factors such as mental health problems, these risk factors are more strongly associated with SSH, and there are risk factors that appear to be specific to NSSH (higher IQ and maternal education) and SSH (lower IQ and parental self-harm; Mars et al., 2014).

Examining their aetiological differences and similarities may offer insights into why NSSH and SSH co-occur and yet differ from each other. Twin studies may offer insights into the potential aetiological difference between NSSH and SSH. In a systematic review of twin studies, based on four large-scale epidemiological twin datasets (Cho, Guo, Iritani, & Hallfors, 2006; Fu et al., 2002; Glowinski et al., 2001; Statham et al., 1998), Voracek and Loibl (2007) reported that heritability estimates for a broader phenotype of suicidal behaviour (suicide thoughts, plans and attempts) ranged from 30% to 55%. A more recent population based twin study not included in the systematic review found that heritability for thoughts of self-harm and suicide ranged from 45% to 74% (Althoff et al., 2012).

However, little empirical attention has been given to the aetiological overlap between NSSH and SSH. One study, based on an Australian twin sample, investigated the shared aetiology between NSSH and suicidal ideation, and found that their heritabilities ranged from 37% to 59%, and the correlation between these two traits was substantially explained by overlapping genetic influences (62–76%; Maciejewski et al., 2014). An unpublished doctoral research was conducted among an American female twin sample, and found that both NSSH and SSH were heritable, with a genetic correlation of 0.45, and a non-shared environmental correlation of 0.09 (Durrett, 2006). However, this twin sample was entirely female, hence the overall heritability estimates of NSSH and SSH, and their genetic correlations in a population with both males and females are still unclear.

The interpersonal theory of suicide also posits that mental health conditions may elevate individuals' risks of developing thwarted belongingness and perceived burdensomeness, subsequently increasing the risk of suicide (Joiner, 2007; Van Orden et al., 2010). Apart from NSSH, experiencing a mental health condition may also increase the risk of acquiring the capability for suicide, e.g. painful experiences of self-starvation for anorexia nervosa (Joiner, 2007; Van Orden et al., 2010). Several twin studies have investigated the aetiological relationships between self-harm-related phenotypes and mental health conditions. For example, shared genetic risk factors were found to explain substantially the comorbidity of suicide attempt with anorexia nervosa and major depressive disorder (MDD) in a Swedish female twin sample, with non-shared environmental factors responsible for the rest of the comorbidity (Thornton, Welch, Munn-Chernoff, Lichtenstein, & Bulik, 2016). A similar pattern was seen among female twins in an Australian twin sample when using a more broadly defined suicidality and eating disorder phenotypes with MDD (Wade, Fairweather-Schmidt, Zhu, & Martin, 2015). In the same Australian twin sample, overlapping genetic and non-shared environmental factors were found to account for the associations between cannabis involvement and suicidal ideation (Delforterie et al., 2015). In an American twin sample, a large proportion of genetic variance was suggested to be shared among suicidal ideation, MDD and conduct disorder, with lower shared environmental influences (Linker, Gillespie, Maes, Eaves, & Silberg, 2012). In the aforementioned Australian twin sample, correlations between high-risk trauma exposure and self-injurious thoughts and behaviours were also found to be influenced by genetic factors in both men and women, and non-shared environmental factors in women only (Richmond-Rakerd et al., 2019).

Among the aforementioned studies which investigated the aetiological relationships between self-harm–related phenotypes and mental health conditions, most of them studied suicidal ideation or suicide attempt only, or a broadly defined suicidality phenotype. One exception is the high-risk trauma exposure study, which included both NSSH and SSH, but these two variables were tied together with suicidal ideation in a common pathway model, and was termed ‘self-injurious thoughts and behaviours’ (Richmond-Rakerd et al., 2019). Hence, it was impossible to know how high-risk trauma exposure was correlated with NSSH and SSH differently, and whether their genetic and environmental correlations were different. This calls for the need to investigate aetiological relationships between a range of mental health problems and both NSSH and SSH, as well as to verify whether such aetiological relationships differ for NSSH *v.* SSH.

To our knowledge, no previous study has investigated the aetiological difference between NSSH and SSH in a population-based twin sample. Hence, using an UK population-based sample of over 9000 twins, the current study (pre-registered: https://osf.io/dy25a) was conducted with the following aims:

1. To investigate to what extent SSH and NSSH share the same genetic and environmental aetiologies; and
2. To investigate the genetic and environmental overlap between a range of mental health problems in adolescence and both lifetime SSH and NSSH reported at age 21 years, as well as to establish whether the patterns of these relationships differ between NSSH and SSH (i.e. to test whether covariances show a differential decomposition in terms of genetic and environmental factors for NSSH *v.* SSH).

We hypothesised that:

1. SSH and NSSH are substantially heritable. There are genetic and environmental correlations between SSH and NSSH, but also unique genetic and environmental components contributing to each facet of self-harm.
2. There are distinct patterns of aetiological relationships between mental health problems and each of NSSH and SSH, e.g. unequal genetic correlations between SSH and mental health problems *v.* NSSH and mental health problems.

## Methods

The preregistration for this study and the code is available on the Open Science Framework: https://osf.io/dy25a

### Participants

Participants were from the Twins Early Development Study (TEDS), a longitudinal twin study following a population sample of twins born between 1994 and 1996 in England and Wales, who were contacted via parents after birth and followed-up across development. At the start of data collection, sample characteristics were close to the characteristics of the UK population as assessed with census data, which remains reasonably the case despite some attrition (Rimfeld et al., 2019). The data were collected in multiple waves across development, by posting questionnaires and test booklets, by telephone, by web-based platforms and at age 21, by a smartphone app. For those preferring questionnaires and test booklets, they have been made available across all data collections. More details about the recruitment process and the sample are described in Rimfeld et al. (2019).

Data used in this study were collected when the twins were 16 (for mental health measures) and 21 years old (for self-harm measures). We applied the standard exclusion criteria in TEDS before carrying out the analyses. Twin pairs with serious medical conditions, missing zygosity and gender information, missing first contact data and extreme perinatal conditions were excluded from the analyses.

After applying exclusion criteria, there are a total of 9063 twins (62.4% female) who answered the self-harm-related questions. The average age of the twins when the questionnaire booklets were completed is 22.3 years (s.d. = 0.91 years), ranging from 20.5 to 25.3 years.

### Measures

#### Self-harm measures

Three questions related to self-harm were used. They were taken and adapted from the Child & Adolescent Self-harm in Europe (CASE) study (Madge et al., 2008). The first question was ‘In your lifetime, have you ever hurt yourself on purpose in any way (e.g. by taking an overdose of pills, or by cutting yourself?)’. Participants chose their responses out of five options, ranging from ‘No’, ‘Yes, once or twice’, ‘Yes, 3–5 times’, ‘Yes, 6–10 times’ to ‘Yes, more than 10 times’.

If the participants answered ‘Yes’ in the first question, they were asked to answer other follow-up questions, which included two questions related to NSSH and SSH. For NSSH, participants were asked, ‘In your lifetime, have you ever hurt yourself on purpose without intending to kill yourself?’. For SSH, participants were asked, ‘In your lifetime, on any of the occasions you have hurt yourself on purpose, have you ever seriously wanted to kill yourself?’. The options available for these two questions were the same as the first question. Of note, participants who did not endorse a history of self-harm in the first question were coded as ‘No’ in the subsequent NSSH and SSH questions.

#### Mental health measures

A total of 19 measures related to mental health were collected at age 16 for this study. They were either reported by the parents, or by the twin themselves. These included internalising problems (anxiety, depression, anxiety sensitivity, insomnia and eating disorder), externalising problems [attention deficit hyperactivity disorder (ADHD), inattention, hyperactivity and impulsivity, and emotional lability], psychotic-like experiences (negative symptoms of schizophrenia, hallucinations, paranoia, hedonia, grandiosity and anhedonia), substance abuse (alcohol abuse, smoking and cannabis use) and others (total difficulties and autistic traits). Details of the measures are summarised in Table 1.

**Table 1.** Measures related to mental health collected at age 16

Category	Phenotypes measured	Questionnaire	Rater	No. of items	Additional information
Others	Total difficulties	Strengths and Difficulties Questionnaire (SDQ; Goodman, 1997)	Twin, parent	12 for parent, 20 for twin	For parents, the measure was derived from three subscales: hyperactivity scale, conduct scale and peer problems scale. For twins, the measure was derived from the emotional scale, peer problems scale, hyperactivity scale and conduct scale. All scales were on the basis of twins’ behaviours over the last 6 months.
	Autistic traits	Abbreviated Autism Spectrum Quotient (AQ; Baron-Cohen, Wheelwright, Skinner, Martin, & Clubley, 2001)	Twin, parent	28 for parent, 13 for twin	Quantifies where an individual is situated on the continuum from autism to normality, assesses symptoms over the last 6 months.
Internalising problems	Anxiety	Anxiety-Related Behaviours Questionnaire (ARBQ; Eley et al., 2003)	Parent	19	Parents rated general distress, separation anxiety, fears, obsessive-compulsive behaviours, and shyness/inhibition of the twins over the last 6 months.
	Depression	Short Mood and Feelings Questionnaire (MFQ; Angold, Costello, Messer, & Pickles, 1995)	Twin, parent	13 for parent, 11 for twin	The parent and child booklet measures included different subsets of the MFQ items, hence the parent and child scales include different numbers of items. Assesses symptoms over the last 2 weeks.
	Sensitivity towards anxiety	Childhood Anxiety Sensitivity Index (CASI; Silverman, Fleisig, Rabian, & Peterson, 1991)	Twin	18	Assesses the belief that anxiety symptoms have negative consequences over the last 6 months.
	Eating disorder	Eating Disorder Diagnostic Scale (EDDS; Stice, Fisher, & Martinez, 2004)	Twin	4	A brief self-report measure for diagnosing anorexia nervosa, bulimia nervosa, and binge eating disorder, assesses symptoms over the last 6 months.
	Insomnia	Insomnia Severity Index (Bastien, Vallières, & Morin, 2001; Morin, 1993)	Twin	7	An instrument to quantify perceived current insomnia severity.
Externalising problems	ADHD	Conners’ Parent Rating Scale (CPRS; Conners, 2001)	Parent	18	Derived from the inattention and hyperactivity/impulsivity subscale. Assesses ADHD symptoms over the last 6 months.
	Inattention	CPRS	Parent	9	CPRS subscale
	Hyperactivity/impulsivity	CPRS	Parent	9	CPRS subscale
	Emotional lability	CPRS	Parent	3	CPRS subscale
	ADHD	Strengths and Weaknesses of ADHD Symptoms and Normal Behaviour Scale (SWAN; Swanson et al., 2012)	Twin	18	Assesses ADHD in a dimensional approach. Twins were asked about their ADHD traits compared to others of their age.
Psychotic-like experiences	Negative symptoms	Specific Psychotic Experiences Questionnaire (SPEQ; Ronald et al., 2014)	Parent	10	Subscale of SPEQ based on Scale for the Assessment of Negative Symptoms (SANS; Andreasen, 1989), assesses negative symptoms expressed by twins.
	Hallucinations	SPEQ	Twin	9	Subscale of SPEQ based on Cardiff Anomalous Perceptual Scale (CAPS; Bell, Halligan, & Ellis, 2006), assesses how frequently twins have hallucination experiences.
	Paranoia	SPEQ	Twin	15	Subscale of SPEQ based on Paranoid Checklist (Freeman et al., 2005), assesses how frequently twins have paranoid thoughts or feelings.
	Hedonia	SPEQ	Twin	10	Subscale of SPEQ based on Temporal Experience of Pleasure Scale (TEPS; (Gard, Gard, Kring, & John, 2006), assesses hedonia over the last month.
	Grandiosity	SPEQ	Twin	8	Grandiosity and delusion subscale of SPEQ, assesses grandiosity thoughts or feelings over the last month.
	Anhedonia	Introvertive Anhedonia Scale (Mason, Linney, & Claridge, 2005)	Twin	7	A subscale in the Oxford-Liverpool Inventory of Feelings and Experiences (O-LIFE) which assesses schizotypy traits.
Substance abuse	Alcohol abuse	‘How often do you have six or more units of alcohol on one occasion?’	Twin	1	Options: ‘Never’, ‘Less than monthly’, ‘Monthly’, ‘Weekly’ and ‘Daily or almost daily’. Missingness in ‘Never’ for twins who never drank alcohol was recoded based on ‘No’ responses in a previously asked question ‘Have you ever drunk alcohol?’
	Smoking	‘How many cigarettes have you smoked, in total, in your lifetime?’	Twin	1	Options: ‘Less than 5’, ‘5–19’, ‘20–49’, ‘50–99’ and ‘100 or more’. Twins who never smoked cigarettes were recoded as ‘0’ based on ‘No’ responses in a previously asked question ‘Have you ever smoked?’
	Cannabis use	‘How many times have you used cannabis?’	Twin	1	Options: ‘Less than 5’, ‘5–19’, ‘20–49’, ‘50–99’ and ‘100 or more’. Twins who never smoked cigarettes were recoded as ‘0’ based on ‘No’ responses in a previously asked question ‘Have you ever tried cannabis?’

*Note:* Substance abuse questions are from a questionnaire on alcohol, tobacco and cannabis use in TEDS, which was adapted from the ‘Life of a 16 + Teenager’ questionnaire from Avon Longitudinal Study of Parents And Child (ALSPAC; Boyd et al., 2013). More details about each measure can be found on TEDS’ data dictionary: https://www.teds.ac.uk/datadictionary/home.htm.

### Analyses

#### Structural equation modelling

Statistical analyses were carried out using the structural equation modelling R package OpenMx (Boker et al., 2011) in the R version 3.6.2 environment (R Core Team, 2017). The classical twin design was employed to decompose the variance of the phenotypes into additive genetic (A), shared environmental (C) and non-shared environmental (E) influences. Additive genetic influences assume the allelic effects are additive but not interactive. Shared environmental influences are environmental factors shared by the twin pairs that make them more similar to each other, whereas non-shared environmental influences include environmental factors unique to each twin and also measurement error. Decomposing the variance into genetic and environmental components is possible due to the shared segregated genes among monozygotic (MZ) twins (100%) and also dizygotic (DZ) twins (50% on average).

Analyses were carried out using the liability threshold model (Falconer, 1965). The ordinal variables are regarded as expressions of underlying continuous liabilities for NSSH and SSH which are normally distributed. For NSSH and SSH, four thresholds were estimated for the continuums of liability, resulting in five observed categories corresponding to the five levels on the ordinal scales. In all models, for ordinal variables, sex and age were included as covariates in the threshold equations. For continuous variables, we adjusted for age and sex by individually regressing each variable on both covariates, and used the residuals in subsequent analyses. Whenever necessary, the residuals of these continuous variables were either log-transformed or cube-rooted to normalise the distributions for compatibility with parametric methods. The 95% confidence intervals (CIs) were computed using the maximum likelihood method. Model comparison was done by means of likelihood ratio chi-squared testing.

### Bivariate model

To explore the first aim of the study, we firstly fitted a bivariate constrained correlation model, with constraints applied whereby the within-twin correlations and the liability thresholds are the same across birth order and zygosity. The constrained correlation model enabled us to derive the phenotypic correlation and also cross-twin cross-trait correlation between NSSH and SSH.

To decompose the phenotypic correlation between NSSH and SSH into genetic and environmental components, we fitted a bivariate ACE model using Cholesky decomposition, which is then transformed into a correlated factors model. These correlations indicate to what extent the genetic and environmental influences on NSSH are the same as the genetic and environmental influences on SSH. To formally test whether these two variables have different aetiologies, we constrained the genetic, shared environmental and non-shared environmental influences of one variable to be the same as another, and tested the goodness of fit of this constrained bivariate ACE model against the unconstrained bivariate ACE model. A graphical representation of the constrained and non-constrained bivariate ACE models is shown in online Supplementary Fig. S1. We also tested for quantitative sex differences in the aetiologies for NSSH and SSH (see online Supplementary Method S1).

#### Trivariate model

For the second aim of the study, trivariate analyses were used to model NSSH, SSH and a mental health measure together. This is similar to the bivariate ACE model but extended to include one extra variable. The cross-twin cross-trait correlations among three variables were estimated to partition the phenotypic correlations into genetic and environmental correlations. In total, we fitted 24 trivariate models out of 19 mental health measures (see Table 1 for details of each variable, in which some are subscales from the same measure). For each trivariate ACE model, we tested whether the pairs of genetic correlations (i.e. *r*_g_ between a measure and NSSH, and *r*_g_ between the measure and SSH), shared and non-shared environmental correlations can be constrained to be the same. This tested whether NSSH and SSH share similar aetiologies with the mental health measures. A graphical representation of the constrained and non-constrained bivariate ACE models is shown in online Supplementary Fig. S2. Due to multiple testing, the *p* values from the comparison tests were corrected with the false discovery rate method, which controls for the expected proportion of false positives among the rejected hypotheses. We set the threshold for statistical significance at a *q* value <0.05 after correcting for multiple testing.

## Results

### Descriptive statistics

Table 2 shows the descriptive statistics for NSSH and SSH in the twin sample. The prevalences for NSSH and SSH in the full sample are 21.9% and 10.5% respectively, with higher prevalences among the female twins (*p* < 0.001 in both cases). The estimated phenotypic correlation between NSSH and SSH in the twin sample is 0.87, as shown in online Supplementary Table S1. Descriptive statistics for the mental health measures are presented in online Supplementary Table S2.

**Table 2.** Descriptive statistics for NSSH and SSH

Phenotype	Frequency	Full sample			Male			Female		
		Total *N*	*N*	%	Total *N*	*N*	%	Total *N*	*N*	%
NSSH	No	9063	7080	78.1	3407	2887	84.7	5656	4193	74.1
	Yes, once or twice		951	10.5		295	8.7		656	11.6
	Yes, 3–5 times		293	3.2		72	2.1		221	3.9
	Yes, 6–10 times		195	2.2		39	1.1		156	2.8
	Yes, more than 10 times		544	6.0		114	3.3		430	7.6
SSH	No	9061	8106	89.5	3407	3143	92.3	5654	4963	87.8
	Yes, once or twice		640	7.1		180	5.3		460	8.1
	Yes, 3–5 times		144	1.6		47	1.4		97	1.7
	Yes, 6–10 times		60	0.7		11	0.3		49	0.9
	Yes, more than 10 times		111	1.2		26	0.8		85	1.5
Mean age (years)		22.3			22.3			22.2		
Number of twins reporting ‘Yes’ for either NSSH or SSH, or both (%)		2057 (22.7%)			542 (15.9%)			1515 (26.8%)		
Number of twins reporting ‘Yes’ for both NSSH and SSH (%)		881 (9.7%)			242 (7.1%)			639 (11.3%)		
Number of twins reporting ‘Yes’ for NSSH only (%)		1102 (12.2%)			278 (8.2%)			824 (14.6%)		
Number of twins reporting ‘Yes’ for SSH only (%)		74 (0.8%)			22 (0.6%)			52 (0.9%)		

### Bivariate models

**Fig. 1.**
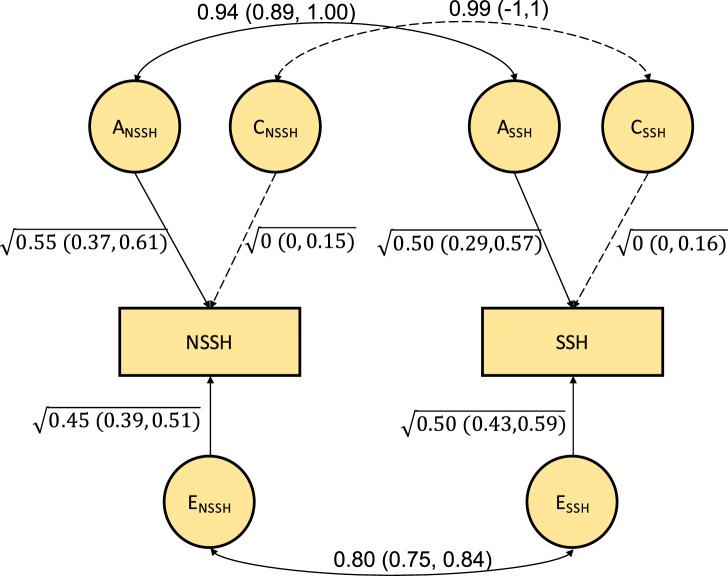
Path diagram of the unconstrained bivariate ACE model for NSSH and SSH.

As shown in online Supplementary Table S1, in the bivariate constrained correlation models, both NSSH and SSH have similar MZ correlations and also DZ correlations. In the bivariate ACE model, both NSSH and SSH have similar genetic architecture. Both NSSH and SSH are under substantial additive genetic influences (55% for NSSH, 50% for SSH) and non-shared environmental influences (45% for NSSH, 50% for SSH), and there was no shared environmental influence, as shown in Fig. 1. The estimates for NSSH and SSH in univariate ACE models are similar to what were estimated in the bivariate ACE model (see online Supplementary Method S2, Note S1 and Table S3).

In terms of the bivariate relationship, the genetic correlation between NSSH and SSH was 0.94. Based on Fig. 1, using path tracing we can derive the phenotypic correlation. This is done by tracing the paths from NSSH to SSH via the correlated latent A or E factors and summing up the products of these chains, i.e. (√0.55 × 0.94 × √0.50) + (√0.45 × 0.80 × √0.50), giving us a phenotypic correlation of 0.87. This means that ~57% of the phenotypic correlation is due to correlated genetic effects i.e. (√0.55 × 0.94 × √0.50)/0.87 = 0.57), and the rest of the correlation (~43%) accounted for by the non-shared environmental influences. As shown in online Supplementary Table S4, in the likelihood ratio test, the constrained bivariate ACE model was not a significantly worse fit than the non-constrained bivariate ACE model (*p* = 0.47), suggesting no aetiological difference between NSSH and SSH. We also found no evidence of quantitative sex differences in the aetiologies of NSSH and SSH (see online Supplementary Note S2, Tables S4–S6).

### Trivariate models

**Fig. 2.**
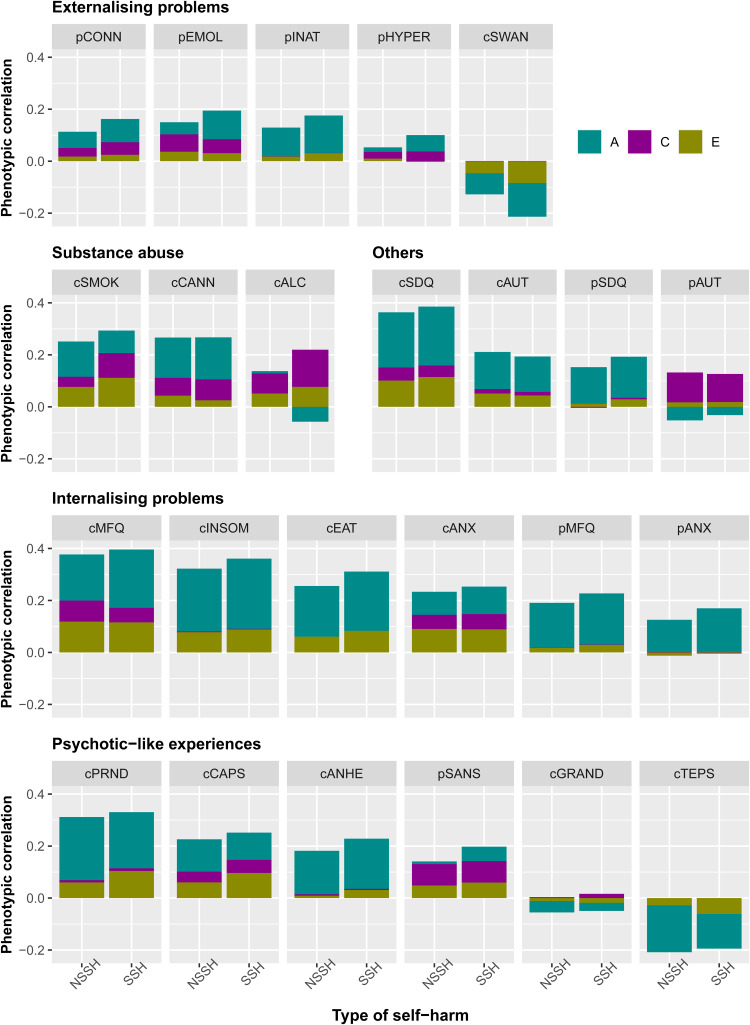
Phenotypic correlations between each mental health measure and NSSH and SSH, and the contributions of A, C and E in the correlations. Measures beginning with letter c = child-rated, p = parent-rated. CONN = ADHD; EMOL = emotional lability; INAT = inattention; HYPER = hyperactivity/impulsivity; SWAN = ADHD symptoms (reversed); SMOK = smoking; CANN = cannabis use; ALC = alcohol; SDQ = total difficulties; AUT = autistic traits; MFQ = depression; INSOM = insomnia; EAT = eating disorder; ANX = anxiety; PRND = paranoia; CAPS = hallucinations; ANHE = anhedonia; SANS = negative symptoms; GRAND = grandiosity; TEPS = hedonia.

Figure 2 shows the phenotypic correlations between each mental health measure with both NSSH and SSH. For NSSH, the measure that is positively correlated with it the most is child-rated MFQ for depressive symptoms, *r* = 0.38, whereas the least positively correlated measure is parent-rated CPRS hyperactivity subscale, *r* = 0.05. The measure that is negatively correlated with NSSH the most is the child-rated TEPS for hedonia, *r* = −0.21, whereas the least negatively correlated measure is child-rated grandiosity subscale from SPEQ, *r* = −0.05.

For SSH, the measure that is most positively correlated with it is also child-rated MFQ, *r* = 0.40, and the least positively correlated measure is parent-rated AQ for autism, *r* = 0.09. The mental health measure that is most negatively correlated with it is the child-rated SWAN for ADHD symptoms, *r* = −0.21, in which a higher SWAN score reflects fewer ADHD symptoms, whereas the least negatively correlated measure is child-rated grandiosity subscale from SPEQ, *r* = −0.03. The strengths of phenotypic correlations between different mental health measures with both NSSH and SSH are visualised in Fig. 2. Numerical results of the phenotypic correlations with 95% CIs, together with the contributions of genetic and environmental factors to the correlations are presented in online Supplementary Table S7. In general, a strikingly similar pattern of results was observed for NSSH *v.* SSH. For explanation for negative contribution to positive phenotypic correlations, see online Supplementary Note S3.

**Fig. 3.**
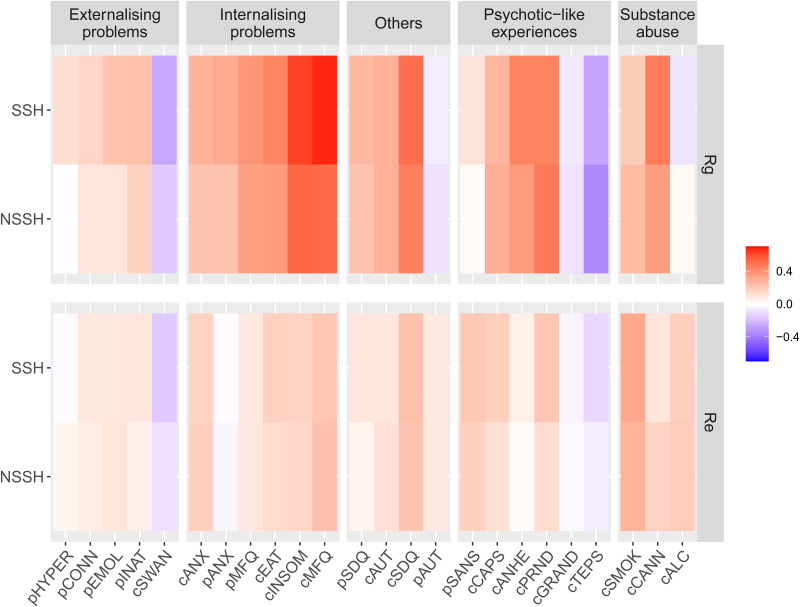
Genetic (*R*_g_) and non-shared environmental (*R*_e_) correlations between each mental health measure and both NSSH and SSH. Measures beginning with letter c = child-rated, p = parent-rated. HYPER = hyperactivity/impulsivity; CONN = ADHD; EMOL = emotional lability; INAT = inattention; SWAN = ADHD symptoms (reversed); ANX = anxiety; MFQ = depression; EAT = eating disorder; INSOM = insomnia; SDQ = total difficulties; AUT = autistic traits; SANS = negative symptoms; CAPS = hallucinations; ANHE = anhedonia; PRND = paranoia; GRAND = grandiosity; TEPS = hedonia; SMOK = smoking; CANN = cannabis use; ALC = alcohol.

In terms of the genetic correlations (*r*_g_), as shown in Fig. 3, for NSSH, the most positive genetic correlation is with the child-rated Insomnia Severity Index, *r*_g_ = 0.53. The measure with the most negative genetic correlation with NSSH is the child-rated TEPS for hedonia, *r*_g_ = −0.36. For SSH, the most positive genetic correlation is with the child-rated MFQ for depressive symptoms, *r*_g_ = 0.67, whereas the most negative genetic correlation is also with the child-rated TEPS, *r*_g_ = −0.28.

For shared environmental correlations (*r*_c_), since both NSSH and SSH are under little shared environmental influences, most of the shared environmental correlations between the mental health measures were not significant, mostly with the pattern of *r*_c_ = 0.99, and 95% CI ranging from −1 to 1.

For non-shared environmental correlations (*r*_e_), for NSSH, the most positive non-shared environmental correlation was with child-reported number of cigarettes used, *r*_e_ = 0.28, whereas the most negative non-shared environmental correlation is with child-rated SWAN scores, *r*_e_ = −0.10. Similarly, for SSH, the most positive and most negative non-shared environmental correlations were with the child-reported number of cigarettes used, *r*_e_ = 0.32, and child-rated SWAN scores, *r*_e_ = −0.16, respectively.

In general, the aetiological relationships between the mental health measures with NSSH do not differ substantially from that of SSH. The numerical results for the *r*_g_, *r*_c_ and *r*_e_ together with their estimated 95% CIs are presented in online Supplementary Table S8. In the likelihood ratio tests, three measures (parent-rated inattention, parent-rated depression and child-rated anhedonia) had their constrained trivariate models being worse than the non-constrained counterparts at nominal significance (*p* = 0.04, *p* = 0.02 and *p* = 0.03 respectively). However, after multiple testing correction, none of the constrained trivariate models was significantly worse than their non-constrained counterparts (see online Supplementary Table S9), suggesting that *r*_g_, *r*_c_ and *r*_e_ values between each mental health measure and self-harm do not vary depending on the type of self-harm, either NSSH or SSH.

## Discussion

### No evidence of aetiological difference between NSSH and SSH

In this study, we found that NSSH and SSH are strongly correlated. They have similar aetiological architectures, with substantial genetic and non-shared environmental influences, but no significant shared environmental influences. The high genetic and non-shared environmental correlations between NSSH and SSH imply that they are influenced by similar biological mechanisms and unique environmental factors, whereas there are very little unique genetic and environmental influences that contribute to each of them specifically. Hence, the first hypothesis is only partially supported. We found no evidence that NSSH and SSH differ in terms of their shared genetic and environmental aetiologies with a range of mental health problems. Hence, the second hypothesis was not supported. There were also no quantitative sex differences in the aetiologies of NSSH and SSH.

Our findings are in line with previous study, in which about half of the variance for SSH (or a more broadly defined suicidality) is explained by genetic influences, with approximately another half explained by non-shared environmental influences, and shared-environmental influences playing little to no role (Althoff et al., 2012; Glowinski et al., 2001). An AE aetiological architecture also underlies NSSH (Durrett, 2006; Maciejewski et al., 2014). Previous study focusing on SSH only demonstrated significant genetic and non-shared environmental correlations between self-harm-related phenotypes and eating disorders, MDD (Thornton et al., 2016; Wade et al., 2015), cannabis use (Delforterie et al., 2015) and high-risk trauma exposure (Richmond-Rakerd et al., 2019). Our study extended those findings by considering SSH and NSSH together with a range of mental health problems.

### Self-harm as a continuum

As we found no evidence of aetiological difference between NSSH and SSH, our findings support the view that the distinction between NSSH and SSH may be a false dichotomy (Kapur et al., 2013), and should be regarded as a continuum, with NSSH and SSH at the opposite ends, and NSSH being a gateway towards SSH (Brausch & Gutierrez, 2010; Linehan, 1986; Stanley et al., 1992). We also showed that NSSH is more prevalent than SSH, which fits the theory, as SSH is at the more severe end of the self-harm continuum and hence is less prevalent. The results also fit the interpersonal theory of suicide, in which NSSH may elevate one's acquired capability for lethal SSH (Joiner, 2007; Van Orden et al., 2010). In addition, previous research found that most adolescents engage in self-harm for more than one reason (Scoliers et al., 2009). The intention of dying during self-harm can be ambivalent (Hawton, Cole, O'Grady, & Osborn, 1982). It can change from episode to episode, or even within a single episode of self-harm (Cooper et al., 2011). Together, these findings question the utility of creating new diagnoses for NSSH and SSH in the DSM-5. However, it is important to note that the phenotypes used in the current study do not entirely fit into the definitions for NSSI and suicidal behaviour disorder in the DSM-5. Specifically, in our study, NSSH includes non-suicidal self-poisoning, but NSSI in DSM-5 is defined as ‘self-inflicted damage to the surface of the body’, which excludes non-suicidal self-poisoning (American Psychiatric Association, 2013).

### Clinical and research implications

The high *r*_g_ and *r*_e_ between NSSH and SSH suggest that for adolescents or young adults who engage in NSSH and progress to SSH, the underlying factors are likely to be influenced by similar biological mechanisms and unique environmental influences, suggesting opportunities to prevent both of them with similar preventative interventions. These shared underlying factors are also likely to be linked with the aetiologies of mental health problems ranging from internalising problems to externalising problems, substance use and psychotic-like experiences. Hence, interventions for these mental health problems may be helpful to address NSSH and SSH too.

The phenotypic correlations estimated between the mental health measures with both NSSH and SSH also suggest the role of mental health problems as risk factors for both NSSH and SSH. The mental health problems were measured at age 16, which is 5 years before lifetime NSSH and SSH questions were asked at age 21, suggesting longitudinal associations between these mental health problems with both NSSH and SSH. Nonetheless, it is also important to be cautious in interpreting these associations, as NSSH and SSH were measured using lifetime-ever items. Participants could therefore have engaged in self-harm prior to age 16.

The current study suggests that conducting research by regarding self-harm as a continuum and regardless of suicide intent may be helpful to acquire novel insights, particularly in investigating its biological mechanisms. This is supported by previous studies, for example, a genome-wide association study (GWAS) that regarded suicidality as a continuum, identified three novel genome-wide significant loci associated with suicidality (Strawbridge et al., 2019). Another GWAS that investigated broad sense self-harm also found a novel genome-wide significant locus associated with self-harm (Campos et al., 2020). This shows that investigating self-harm regardless of motivation could be valuable to gain biological insights into the liability of both self-harm and suicidality. The current study also shows that self-harm is a complex dimensional trait associated with a range of mental health problems. This is consistent with the Research Domain Criteria approach with emphasises transdiagnostic dimensions (Insel et al., 2010).

### Strengths and limitations

One of the strengths in this study is that the self-harm measures are ordinal variables with multiple levels, instead of being binary variables with ‘Yes’ or ‘No’ responses. This yielded more statistical power. Another strength is the inclusion of parent-reported mental health measures, and that findings appear to be robust across informants. Having both male and female twins in this sample also enabled us to obtain the overall A, C and E estimates in the population, rather than only for a specific gender.

The limitations in the current study are mostly related to the classical twin design assumptions. One of them is the generalisability of twin research findings to the general population which are mostly singletons. However, the prevalence of lifetime NSSH (21.9%) and SSH (10.5%) in the current study seem to be comparable with the prevalence of lifetime NSSH and SSH in the 16–24 years age group in the Adult Psychiatric Morbidity Survey (17.5% and 9.0% respectively) conducted in England (Mcmanus et al., 2016). Another assumption in the classical twin design is the absence of gene–environment interaction and correlation (Rijsdijk & Sham, 2002), which may have led to bias of estimates in the current study.

Moreover, NSSH and SSH in this study were not measured at two separate times, hence their temporal relationship should be interpreted with caution. The stronger associations between self-rated mental health measures and self-reported self-harm may be due to shared-method variance (i.e. variance attributed to the measurement method rather than to the constructs represented by the measures; Podsakoff, MacKenzie, Lee, & Podsakoff, 2003). Whenever possible, we included parental reports of similar mental health measure, so that we could compare the estimates derived from parent-rated and self-rated measures. However, this was not possible for all mental health measures, and is hence a limitation of the current study.

Using classical twin design, we only estimated correlations but not causal estimates. Hence, the causal relationships between the mental health problems with self-harm could not be determined here. However, recent findings using polygenic scores and Mendelian randomisation have shown that some of these mental health problems likely play a causal role in the aetiology of self-harm (Lim et al., 2020). Future genetically informed studies that integrate polygenic scores and family-based designs can further strengthen causal inference and our understanding of the aetiology of self-harm (Pingault et al., 2018). Furthermore, it is important to note that the study sample consisted of young adults, hence generalisation to older population should be done with caution, as suicidal behaviour and self-harm are manifested differently in different age groups (Mcmanus et al., 2016, 2019). For the final limitation, we found that among those who reported to have self-harmed (regardless of intention), 11% of them reported to have never engaged in NSSH or SSH. As such, these individuals were not included in our analyses as we could not identify them to either have engaged in NSSH or SSH. This possibly contributed to a loss of statistical power due to the reduction in sample size. The reason for this may be due to the wording in the SSH question (‘Have you ever seriously wanted to kill yourself?’) as the word ‘seriously’ may have influenced those with ambivalent suicidal intent to answer ‘No’ in both NSSH and SSH questions. This also suggests that suicidal intent is fluid and difficult to be captured, and hence categorising self-harm into NSSH and SSH may be a potential threat in clinical practice as it may accidentally exclude those with ambivalent suicidal intent.

## Conclusion

In conclusion, using a UK population-based twin sample, we found evidence for substantial overlap between lifetime NSSH and SSH at both phenotypic and aetiological levels among young adults. Among these young people, both NSSH and SSH also share similar aetiologies with a range of mental health problems. Together, the evidence suggests that among young adults at least, NSSH and SSH should be conceptualised as different ends of the same continuum, rather than two distinct categories.

## Supplementary material

For supplementary material accompanying this paper visit https://doi.org/10.1017/S0033291721000040. click here to view supplementary material
