# Supplementary material for: Investigating the genetic and environmental aetiologies of non-suicidal and suicidal self-harm: a twin study
Source: Psychol Med. 2021 Feb 9;52(15):3391–401. doi: 10.1017/S0033291721000040 (PMC9772908; doi:10.1017/S0033291721000040)
Supplement: Supplementary file 1 [file S0033291721000040sup001.docx]

**Investigating the genetic and environmental aetiologies of non-suicidal and suicidal self-harm: A twin study**

Supplementary material

[Figure S1. Constrained and non-constrained bivariate ACE models 2](#_Toc60754917)

[Method S1. Sex differences analyses 3](#_Toc60754918)

[Figure S2. Constrained and non-constrained trivariate ACE models 4](#_Toc60754919)

[Table S1. Bivariate constrained correlation models for NSSH and SSH. 5](#_Toc60754920)

[Table S2. Descriptive statistics for mental health measures collected at age 16 6](#_Toc60754921)

[Method S2. Univariate analyses 7](#_Toc60754922)

[Note S1. Results of univariate analyses 7](#_Toc60754923)

[Table S3. Univariate models for NSSH and SSH. 8](#_Toc60754924)

[Table S4. Model comparisons for constrained models versus non-constrained models, and sex homogeneity models versus sex heterogeneity models. 9](#_Toc60754925)

[Note S2. Results for sex difference analyses 10](#_Toc60754926)

[Table S5. Polychoric correlations for NSSH and SSH in groups of different zygosity and sex. 11](#_Toc60754927)

[Table S6. ACE estimates from the homogeneity and heterogeneity models for sex differences. 12](#_Toc60754928)

[Table S7. Phenotypic correlations between each measure with NSSH and SSH in the trivariate ACE models, and the contributions of A, C and E to the correlations. 13](#_Toc60754929)

[Note S3. Negative contributions to positive phenotypic correlations 15](#_Toc60754930)

[Table S8. Genetic and environmental correlations between each measure with both NSSH and SSH. 16](#_Toc60754931)

[Table S9. Goodness of fit tests in trivariate analyses. 18](#_Toc60754932)

# Figure S1. Constrained and non-constrained bivariate ACE models


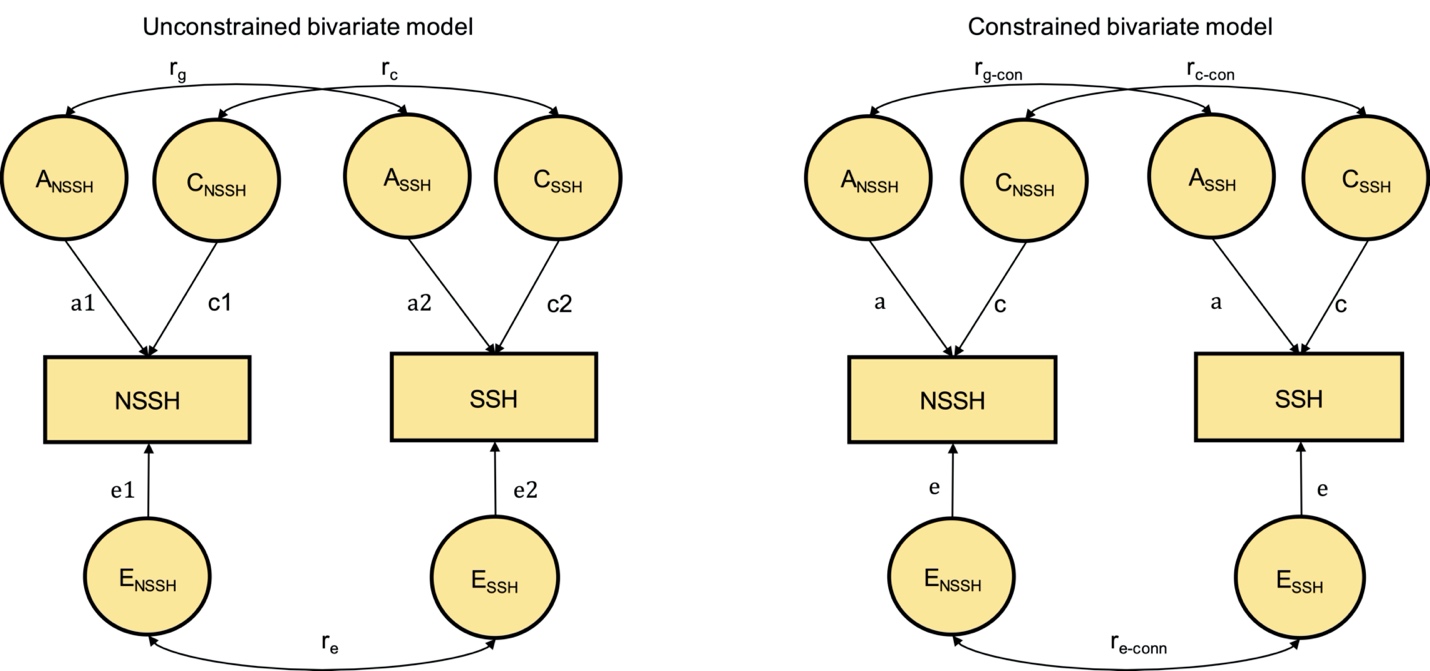


Figure S1. Graphical representation of the unconstrained and constrained bivariate ACE models for NSSH and SSH. In the constrained model, the pairs of *a*, *c* and *e* paths (a1 and a2, c1 and c2, e1 and e2 in the unconstrained model) were constrained to be the same. We then compared the goodness of fit of the constrained model against the unconstrained model. The genetic correlations (r_g_ and r_g-con_), shared environmental correlations (r_c_ and r_c-con_) and non-shared environmental correlations (r_e_ and r_e-con_) in the constrained and unconstrained models were labelled differently to indicate that they are free parameters which can differ in the two models.

# Method S1. Sex differences analyses

We tested for quantitative sex differences by estimating parameters separately for males and females in univariate models for NSSH and SSH. For each phenotype, we fitted univariate constrained correlation models to obtain the MZ and DZ correlations for the same-sex (male and female) and opposite-sex twins separately. We then fitted heterogeneity sex models for NSSH and SSH, in which the A, C and E components of males and females were allowed to be different. Finally, we fitted homogeneity sex models, in which the A, C and E estimates were constrained to be the same for males and females. We compared the goodness of fit between the homogeneity and heterogeneity models. If the homogeneity model does not have a significantly worse fit than the heterogeneity model, this suggests the absence of quantitative sex difference.

# Figure S2. Constrained and non-constrained trivariate ACE models


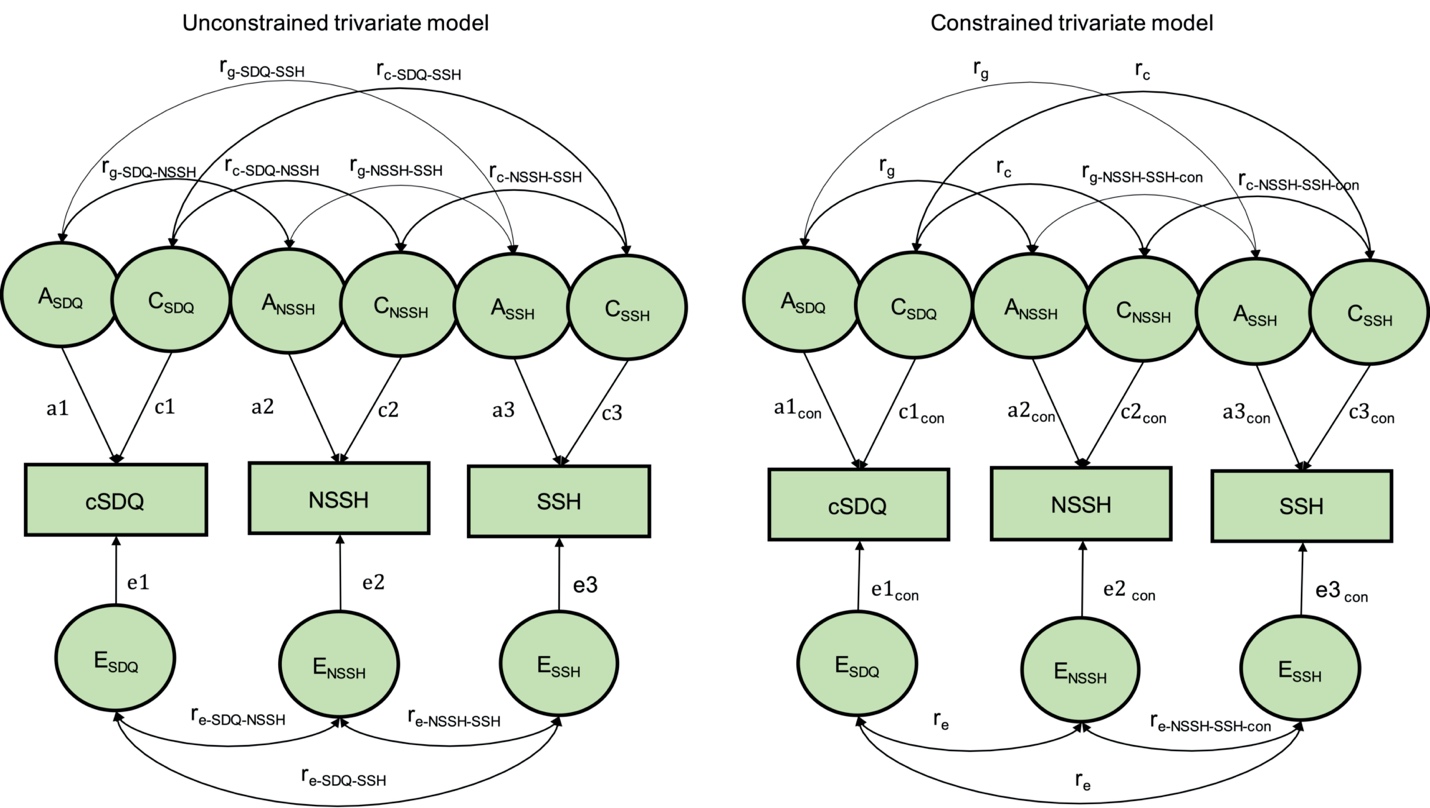


Figure S2. Graphical representation of an unconstrained and constrained trivariate ACE model, using child-rated SDQ (cSDQ) as an example. In the constrained models, the pairs of r_g_ ( r_g-SDQ-SSH_ and r_g-SDQ-NSSH_ ), r_c_ (r_c-SDQ-SSH_ and r_c-SDQ-NSSH_), and r_e_ (r_e-SDQ-SSH_ and r_e-SDQ-NSSH_) were constrained to be the same. We then compared the goodness of fit of the constrained models against the unconstrained model. The a paths (e.g. a1 and a1_con_), c paths (e.g. c1 and c1_con_) and e paths (e.g. e1 and e1_con_) in the unconstrained and constrained models were labelled differently to indicate that they are free parameters which can differ in the two models. This also applies to the genetic (r_g-NSSH-SSH_ and r_g-NSSH-SSH-con_), shared environmental (r_c-NSSH-SSH_ and r_c-NSSH-SSH-con_) and non-shared environmental (r_e-NSSH-SSH_ and r_e-NSSH-SSH-con_) correlations between NSSH and SSH in both models.

# Table S1. Bivariate constrained correlation models for NSSH and SSH.

| Phenotype | NSSH | SSH |
| --- | --- | --- |
| Constrained correlation model (95% CI) |  |  |
| MZ cross-twin correlation | 0.55 (0.49, 0.61) | 0.51 (0.41, 0.59) |
| DZ cross-twin correlation | 0.27 (0.20, 0.34) | 0.22 (0.12, 0.31) |
| MZ cross-twin cross-trait correlation | 0.49 (0.42, 0.55) | |
| DZ cross-twin cross-trait correlation | 0.25 (0.18, 0.32) | |
| Phenotypic cross-trait correlation | 0.87 (0.86, 0.88) | |

# Table S2. Descriptive statistics for mental health measures collected at age 16

| Category | Measure | Sample size | Mean | SD |
| --- | --- | --- | --- | --- |
| Externalising problems | cSWAN | 1474 | 4.81 | 0.82 |
|  | pCONN | 6658 | 5.93 | 6.65 |
|  | pEMOL | 6661 | 1.17 | 1.72 |
|  | pHYPER | 6656 | 2.31 | 3.15 |
|  | pINAT | 6657 | 3.62 | 4.40 |
| Internalising problems | cANX | 6666 | 8.20 | 5.91 |
|  | cEAT | 1465 | 3.24 | 1.92 |
|  | cINSOM | 5175 | 3.80 | 4.12 |
|  | cMFQ | 6665 | 3.74 | 4.51 |
|  | pANX | 6666 | 3.59 | 4.17 |
|  | pMFQ | 6663 | 0.93 | 2.21 |
| Others | cAUT | 6659 | 12.12 | 5.78 |
|  | cSDQ | 6661 | 9.35 | 5.10 |
|  | pAUT | 6665 | 23.75 | 10.76 |
|  | pSDQ | 6669 | 3.46 | 2.99 |
| Psychotic-like experiences | cANHE | 6657 | 1.25 | 1.30 |
|  | cCAPS | 6666 | 4.74 | 6.08 |
|  | cGRAND | 6662 | 5.14 | 4.33 |
|  | cPRND | 6663 | 12.26 | 10.65 |
|  | cTEPS | 6667 | 33.89 | 7.77 |
|  | pSANS | 6660 | 2.68 | 3.79 |
| Substance abuse | Ordinal variables | Sample size | Proportion of twins who at least had taken alcohol (more than 6 units)/cannabis/cigarettes once in their lifetime | |
|  | cALC | 4451 | 8.34% | |
|  | cCANN | 5026 | 20.03% | |
|  | cSMOK | 5007 | 35.34% | |

# Method S2. Univariate analyses

For each phenotype, we firstly fitted a constrained correlation model, whereby both MZ and DZ twin polychoric tetrachoric correlations were estimated. To estimate the relative contributions of additive genetic (A), shared environment (C) and non-shared environment (E) influences on both NSSH and SSH, we fitted a standard ACE genetic model.

# Note S1. Results of univariate analyses

In univariate analyses, as shown in Table S3, fitted saturated model showed that the MZ correlations for NSSH and SSH were 0.54 (95% CI: 0.48, 0.60) and 0.49 (95% CI: 0.39, 0.59) respectively, whereas the DZ correlations were 0.27 (95% CI: 0.20, 0.34) and 0.24 (95% CI: 0.13, 0.34), respectively.

In the univariate ACE model, the additive genetic (A) explains 54% of the variance for NSSH and 49% of the variance for SSH. Shared environmental factors (C) were estimated to explain 0% of the variances in both NSSH and SSH. For non-shared environmental factors (E), 46% and 51% of the variances were explained in NSSH and SSH respectively. Separate ACE model estimates for males and females are reported in Table S6.

# Table S3. Univariate models for NSSH and SSH.

| Phenotype | NSSH (95% CI) | SSH (95% CI) |
| --- | --- | --- |
| Constrained correlation model |  |  |
| MZ cross-twin correlation | 0.54 (0.48, 0.60) | 0.49 (0.39, 0.59) |
| DZ cross-twin correlation | 0.27 (0.20, 0.34) | 0.24 (0.13, 0.34) |
| ACE model |  |  |
| A | 0.54 (0.35, 0.60) | 0.49 (0.22, 0.58) |
| C | 0 (0, 0.16) | 0 (0, 0.21) |
| E | 0.46 (0.40, 0.52) | 0.51 (0.42, 0.60) |
| Covariates (effects on threshold) |  |  |
| Age | 0.04 (0, 0.07) | 0.02 (-0.02, 0.06) |
| Sex | 0.40 (0.33, 0.46) | 0.26 (0.18, 0.33) |

# Table S4. Model comparisons for constrained models versus non-constrained models, and sex homogeneity models versus sex heterogeneity models.

| Model | Description | ep | -2LL | df | AIC | Compared with model | diffLL | Diffdf | p-value |
| --- | --- | --- | --- | --- | --- | --- | --- | --- | --- |
| 1 | Bivariate ACE model | 23 | 18857.03 | 18109 | -17361 | - | - | - | - |
| 2 | Constrained bivariate ACE model | 20 | 18859.58 | 18112 | -17364 | 1 | 2.55 | 3 | 0.47 |
| 3 | NSSH sex heterogeneity ACE model | 18 | 13948.13 | 9049 | -4150 | - | - | - | - |
| 4 | NSSH sex homogeneity ACE model | 15 | 13952.25 | 9052 | -4152 | 3 | 4.12 | 3 | 0.25 |
| 5 | SSH sex heterogeneity ACE model | 18 | 7829.44 | 9049 | -10269 | - | - | - | - |
| 6 | SSH sex homogeneity ACE model | 15 | 7837.31 | 9052 | -10267 | 5 | 7.86 | 3 | 0.05 |

# Note S2. Results for sex difference analyses

The cross-twin within-trait polychoric correlations for NSSH and SSH across different zygosity and sex are presented in Table S5. We found no quantitative sex differences in the aetiology of NSSH and SSH. The heritability and environmental estimates for males and females in the homogeneity and heterogeneity models for each phenotype are presented in Table S6. In the likelihood ratio chi-square tests (see Table S4), for NSSH, the homogeneity sex model did not have a significantly worse fit than the heterogeneity sex model (*p* = 0.25); for SSH, the drop in goodness of fit for the homogeneity sex model was marginally significant (*p* = 0.05).

# Table S5. Polychoric correlations for NSSH and SSH in groups of different zygosity and sex.

| Zygosity and sex | NSSH | | SSH | |
| --- | --- | --- | --- | --- |
|  | N | Cross-twin correlations (95% CI) | N | Cross-twin correlations (95% CI) |
| MZ male | 490 pairs | 0.51 (0.37,0.63) | 490 pairs | 0.70 (0.53,0.81) |
| DZ male | 395 pairs | 0.25 (0.04,0.44) | 394 pairs | 0.29 (0.03,0.53) |
| MZ female | 984 pairs | 0.56 (0.48,0.63) | 986 pairs | 0.42 (0.28,0.53) |
| DZ female | 791 pairs | 0.35 (0.24,0.45) | 792 pairs | 0.28 (0.13,0.42) |
| DZ opposite sex | 1093 pairs | 0.20 (0.09,0.31) | 1094 pairs | 0.17 (0.00,0.34) |
| Single twins | 1557 | - | 1549 | - |
| Total N twins | 9063 | - | 9061 | - |

Note. The correlations and 95% CI were estimated in separate constrained correlated models for NSSH and SSH.

# Table S6. ACE estimates from the homogeneity and heterogeneity models for sex differences.

| Type of self-harm | NSSH | | SSH | |
| --- | --- | --- | --- | --- |
| Estimates from ACE models | Homogeneity model | Heterogeneity model | Homogeneity model | Heterogeneity model |
| A (95% CI) | | | | |
| Male | 0.54 (0.35,0.60) | 0.50 (0,0.62) | 0.49 (0.22, 0.58) | 0.68 (0.29, 0.80) |
| Female |  | 0.41 (0.17,0.61) |  | 0.26 (0,0.51) |
| C (95% CI) | | | | |
| Male | 0 (0,0.15) | 0.01 (0,0.37) | 0 (0,0.21) | 0.01 (0,0.38) |
| Female |  | 0.14 (0,0.57) |  | 0.16 (0,0.42) |
| E (95% CI) | | | | |
| Male | 0.46 (0.40,0.52) | 0.49 (0.38,0.63) | 0.51 (0.42,0.60) | 0.31 (0.20, 0.47) |
| Female |  | 0.44 (0.37,0.52) |  | 0.59 (0.47,0.99) |

# Table S7. Phenotypic correlations between each measure with NSSH and SSH in the trivariate ACE models, and the contributions of A, C and E to the correlations.

| Category | Measure | Type of self-harm | Phenotypic correlation (95% CI) | correlation due to A, RPhA | correlation due to C, RPhC | correlation due to E, RPhE |
| --- | --- | --- | --- | --- | --- | --- |
| Externalising problems | cSWAN | NSSH | -0.127 (-0.202,-0.049) | -0.081 | 0.001 | -0.046 |
|  |  | SSH | -0.213 (-0.305,-0.113) | -0.13 | 0.001 | -0.084 |
|  | pCONN | NSSH | 0.113 (0.077,0.149) | 0.062 (55.1%) | 0.033 (29.2%) | 0.018 (15.7%) |
|  |  | SSH | 0.163 (0.119,0.205) | 0.089 (55.0%) | 0.049 (29.9%) | 0.024 (15.1%) |
|  | pEMOL | NSSH | 0.150 (0.116,0.182) | 0.047 (31.1%) | 0.067 (44.6%) | 0.036 (24.3%) |
|  |  | SSH | 0.195 (0.155,0.234) | 0.110 (56.6%) | 0.054 (27.7%) | 0.031 (15.8%) |
|  | pHYPER | NSSH | 0.053 (-0.029,0.082) | 0.018 (33.1%) | 0.026 (48.9%) | 0.010 (18.1%) |
|  |  | SSH | 0.097 (0.054,0.138) | 0.063 | 0.037 | -0.003 |
|  | pINAT | NSSH | 0.129 (0.093,0.166) | 0.110 (85.5%) | 0.001 (0.8%) | 0.018 (13.7%) |
|  |  | SSH | 0.176 (0.133,0.221) | 0.144 (81.9%) | 0.001 (0.5%) | 0.031 (17.6%) |
| Internalising problems | cANX | NSSH | 0.233 (0.202,0.264) | 0.089 (38.0%) | 0.054 (23.2%) | 0.091 (38.8%) |
|  |  | SSH | 0.253 (0.215,0.289) | 0.106 (41.8%) | 0.059 (23.1%) | 0.089 (35.1%) |
|  | cEAT | NSSH | 0.256 (0.023,0.322) | 0.195 (76.3%) | 0.000 (0.0%) | 0.061 (23.7%) |
|  |  | SSH | 0.311 (0.220,0.391) | 0.228 (73.3%) | 0.000 (0.0%) | 0.083 (26.7%) |
|  | cINSOM | NSSH | 0.322 (0.289,0.357) | 0.242 (75.1%) | 0.003 (0.8%) | 0.078 (24.1%) |
|  |  | SSH | 0.361 (0.322,0.398) | 0.270 (74.8%) | 0.002 (0.6%) | 0.089 (24.6%) |
|  | cMFQ | NSSH | 0.377 (0.348,0.406) | 0.177 (47.1%) | 0.081 (21.5%) | 0.119 (31.4%) |
|  |  | SSH | 0.396 (0.360,0.431) | 0.225 (56.7%) | 0.056 (14.1%) | 0.116 (29.2%) |
|  | pANX | NSSH | 0.113 (0.063,0.162) | 0.124 | 0.001 | -0.013 |
|  |  | SSH | 0.165 (0.108,0.220) | 0.169 | 0.001 | -0.005 |
|  | pMFQ | NSSH | 0.191 (0.176,0.244) | 0.170 (89.3%) | 0.002 (0.8%) | 0.019 (9.9%) |
|  |  | SSH | 0.227 (0.174,0.282) | 0.197 (86.7%) | 0.001 (0.5%) | 0.029 (12.9%) |
| Others | cAUT | NSSH | 0.211 (0.178,0.243) | 0.143 (67.6%) | 0.017 (8.2%) | 0.051 (24.2%) |
|  |  | SSH | 0.194 (0.154,0.232) | 0.136 (70.4%) | 0.014 (7.1%) | 0.044 (22.5%) |
|  | cSDQ | NSSH | 0.364 (0.334,0.393) | 0.213 (58.4%) | 0.050 (13.8%) | 0.101 (27.7%) |
|  |  | SSH | 0.386 (0.351,0.419) | 0.227 (58.7%) | 0.044 (11.5%) | 0.115 (29.7%) |
|  | pAUT | NSSH | 0.080 (0.044,0.115) | -0.052 | 0.115 | 0.017 |
|  |  | SSH | 0.094 (0.052,0.137) | -0.032 | 0.108 | 0.018 |
|  | pSDQ | NSSH | 0.149 (0.114,0.183) | 0.141 | -0.004 | 0.011 |
|  |  | SSH | 0.193 (0.151,0.233) | 0.158 (82.0%) | 0.006 (3.0%) | 0.029 (15.0%) |
| Psychotic-like experiences | cANHE | NSSH | 0.182 (0.149,0.214) | 0.167 (91.9%) | 0.006 (3.2%) | 0.009 (4.9%) |
|  |  | SSH | 0.229 (0.190,0.267) | 0.193 (84.5%) | 0.005 (2.0%) | 0.031 (13.5%) |
|  | cCAPS | NSSH | 0.226 (0.194,0.257) | 0.124 (55.0%) | 0.042 (18.4%) | 0.060 (26.6%) |
|  |  | SSH | 0.252 (0.214,0.288) | 0.105 (41.6%) | 0.051 (20.1%) | 0.096 (38.3%) |
|  | cGRAND | NSSH | -0.052 (-0.087,-0.017) | -0.044 | 0.004 | -0.011 |
|  |  | SSH | -0.034 (-0.075,0.008) | -0.032 | 0.016 | -0.018 |
|  | cPRND | NSSH | 0.312 (0.281,0.342) | 0.243 (78.0%) | 0.009 (2.8%) | 0.060 (19.2%) |
|  |  | SSH | 0.331 (0.293,0.366) | 0.216 (65.4%) | 0.010 (3.1%) | 0.104 (31.5%) |
|  | cTEPS | NSSH | -0.208 (-0.240,-0.176) | -0.180 (86.6%) | 0.000 (0.0%) | -0.028 (13.4%) |
|  |  | SSH | -0.194 (-0.232,-0.157) | -0.133 (68.6%) | 0.000 (0.0%) | -0.061 (31.4%) |
|  | pSANS | NSSH | 0.141 (0.105,0.176) | 0.010 (7.3%) | 0.082 (58.4%) | 0.048 (34.3%) |
|  |  | SSH | 0.198 (0.156,0.239) | 0.056 (28.1%) | 0.083 (41.8%) | 0.060 (30.1%) |
| Substance abuse | cALC | NSSH | 0.138 (0.087,0.189) | 0.009 (6.7%) | 0.077 (56.1%) | 0.051 (37.2%) |
|  |  | SSH | 0.162 (0.107,0.219) | -0.057 | 0.143 | 0.077 |
|  | cCANN | NSSH | 0.267 (0.203,0.330) | 0.155 (58.2%) | 0.068 (25.7%) | 0.043 (16.1%) |
|  |  | SSH | 0.267 (0.196,0.338) | 0.162 (60.5%) | 0.081 (30.1%) | 0.025 (9.4%) |
|  | cSMOK | NSSH | 0.251 (0.202,0.301) | 0.136 (54.0%) | 0.039 (15.5%) | 0.077 (30.5%) |
|  |  | SSH | 0.293 (0.237,0.348) | 0.087 (29.6%) | 0.095 (32.5%) | 0.111 (37.9%) |

# Note S3. Negative contributions to positive phenotypic correlations

For some positive phenotypic correlations, negative contributions from genetic (rPhA) or non-shared environment influences (rPhE) were observed. This is due to negative r_g_ or r_e_ between the traits, which was indicated by the pattern of MZ and DZ cross-twin cross trait correlations. Taking the phenotypic correlation (rPh) between cALC and SSH as example, although the rPh is 0.16, the r_g_ is estimated to be negative. The MZ and DZ ratio of the cross-twin cross-trait correlations between cALC and SSH is 1: 1 since the correlations 0.09 versus 0.11 are nor significantly different, suggesting that the estimated familial covariance between cALC and SSH is mainly due to C and not due to A. This will naturally push the estimate of the r_c_ to be close to 1, and the r_g_ to be close to 0. With the high r_c_, the contribution of the phenotypic correlation due to C (rPhC) was estimated to be 0.14, which was higher than the actual observed cross-twin cross-trait correlations (0.09 and 0.11). The only way to reduce the phenotypic correlations is if the other familial pathway (rPhA, i.e. phenotypic correlation due to A) is negative so that the sum is 0.09 for MZ twins and 0.11 for DZ twins. This causes the r_g_ to be estimated to be negative and rPhA to be estimated to be -0.05. In this way, the sum of the estimated rPhA and rPhC will match the MZ cross-twin cross-trait correlation (-0.057+0.143=0.09). For DZ cross-twin cross-trait correlation of 0.11, it is the sum of the estimated rPhC and half of the rPhA (0.143+(-0.057/2) = 0.11) as DZ twins only share half of their segregated genes on average. Details of rPhA, rPhC and rPhE are in Table S7. The negative contributions for phenotypic correlations found in this study may be sample specific and similar research using other samples can be conducted to replicate the findings.

# Table S8. Genetic and environmental correlations between each measure with both NSSH and SSH.

| Category | Measures | Type of self-harm | Genetic correlation (95% CI) | Shared environmental correlation (95% CI) | Non-shared environmental correlation (95% CI) |
| --- | --- | --- | --- | --- | --- |
| Externalising problems | cSWAN | NSSH | -0.159 (-0.402,0.112) | 1.000 (-1.000,1.000) | -0.096 (-0.266,0.086) |
|  |  | SSH | -0.268 (-0.698,-0.268) | 1.000 (-1.000,1.000) | -0.163 (-0.359,0.048) |
|  | pCONN | NSSH | 0.099 (0.083,0.233) | 1.000 (-0.012,1.000) | 0.069 (-0.019,0.155) |
|  |  | SSH | 0.153 (0.010,0.314) | 1.000 (0.562,1.000) | 0.089 (-0.014,0.177) |
|  | pEMOL | NSSH | 0.098 (0.068,0.224) | 0.868 (-1.000,1.000) | 0.100 (0.022,0.134) |
|  |  | SSH | 0.221 (0.027,0.319) | 0.964 (-1.000,1.000) | 0.089 (-0.023,0.172) |
|  | pHYPER | NSSH | 0.003 (-0.028,0.033) | 0.470 (-1.000,1.000) | 0.034 (-0.069,0.139) |
|  |  | SSH | 0.121 (-0.023,0.267) | 1.000 (-1.000,1.000) | -0.011 (-0.113,0.087) |
|  | pINAT | NSSH | 0.169 (0.086,0.250) | 0.979 (-1.000,1.000) | 0.058 (-0.027,0.142) |
|  |  | SSH | 0.231 (-0.952,0.964) | 0.542 (-1.000,1.000) | 0.096 (-0.004,0.196) |
| Internalising problems | cANX | NSSH | 0.217 (0.105,0.375) | 1.000 (-1.000,1.000) | 0.172 (0.096,0.246) |
|  |  | SSH | 0.279 (0.073,0.487) | 1.000 (-1.000,1.000) | 0.159 (0.071,0.246) |
|  | cEAT | NSSH | 0.352 (0.122,1.000) | 0.668 (-1.000,1.000) | 0.135 (-0.028,0.294) |
|  |  | SSH | 0.434 (0.189,0.817) | 0.831 (-1.000,1.000) | 0.173 (-0.024,0.363) |
|  | cINSOM | NSSH | 0.530 (0.530,0.714) | 1.000 (-1.000,1.000) | 0.148 (0.068,0.227) |
|  |  | SSH | 0.621 (0.621,0.897) | 1.000 (-1.000,1.000) | 0.159 (0.065,0.252) |
|  | cMFQ | NSSH | 0.515 (0.515,0.833) | 0.921 (0.190,1.000) | 0.225 (0.145,0.300) |
|  |  | SSH | 0.671 (0.671,0.982) | 0.873 (0.768,1.000) | 0.207 (0.112,0.301) |
|  | pANX | NSSH | 0.217 (0.016,0.983) | 1.000 (-1.000,1.000) | -0.029 (-0.160,0.104) |
|  |  | SSH | 0.310 (0.114,0.577) | 1.000 (-1.000,1.000) | -0.011 (-0.153,0.130) |
|  | pMFQ | NSSH | 0.343 (-1.000,0.531) | 0.966 (-0.983,1.000) | 0.087 (-0.061,0.185) |
|  |  | SSH | 0.381 (0.264,0.417) | -0.986 (-1.000,1.000) | 0.077 (-0.013,0.220) |
| Others | cAUT | NSSH | 0.287 (0.258,0.402) | 1.000 (-1.000,1.000) | 0.107 (0.030,0.141) |
|  |  | SSH | 0.285 (0.211,0.432) | 1.000 (-1.000,1.000) | 0.086 (-0.004,0.176) |
|  | cSDQ | NSSH | 0.452 (0.419,0.578) | 1.000 (-1.000,1.000) | 0.211 (0.137,0.284) |
|  |  | SSH | 0.502 (0.484,0.694) | 1.000 (0.995,1.000) | 0.228 (0.138,0.315) |
|  | pAUT | NSSH | -0.091 (-0.226,0.029) | 1.000 (1.000,1.000) | 0.079 (-0.004,0.162) |
|  |  | SSH | -0.059 (-0.235,0.088) | 1.000 (0.912,1.000) | 0.078 (-0.018,0.173) |
|  | pSDQ | NSSH | 0.218 (0.096,0.245) | -0.036 (-1.000,1.000) | 0.039 (-0.048,0.123) |
|  |  | SSH | 0.257 (-0.488,0.346) | 0.451 (-1.000,1.000) | 0.094 (-0.007,0.189) |
| Psychotic-like experiences | cANHE | NSSH | 0.365 (-0.975,0.499) | 1.000 (-1.000,1.000) | 0.016 (-0.060,0.095) |
|  |  | SSH | 0.441 (0.441,0.628) | 1.000 (-1.000,1.000) | 0.056 (-0.035,0.147) |
|  | cCAPS | NSSH | 0.297 (0.297,0.574) | 0.965 (0.718,1.000) | 0.118 (0.036,0.196) |
|  |  | SSH | 0.268 (0.268,0.588) | 0.996 (-1.000,1.000) | 0.178 (0.083,0.269) |
|  | cGRAND | NSSH | -0.087 (-0.283,0.104) | 1.000 (-1.000,1.000) | -0.024 (-0.109,0.061) |
|  |  | SSH | -0.067 (-0.337,0.146) | 1.000 (-1.000,1.000) | -0.036 (-0.137,0.066) |
|  | cPRND | NSSH | 0.476 (0.476,0.704) | 0.572 (-1.000,1.000) | 0.127 (0.044,0.206) |
|  |  | SSH | 0.445 (0.445,0.693) | 0.703 (-1.000,1.000) | 0.209 (0.113,0.300) |
|  | cTEPS | NSSH | -0.355 (-0.512,-0.239) | -0.913 (-1.000,1.000) | -0.057 (-0.131,0.018) |
|  |  | SSH | -0.277 (-0.470,-0.135) | -0.662 (-1.000,1.000) | -0.118 (-0.205,0.144) |
|  | pSANS | NSSH | 0.018 (-0.106,0.153) | 1.000 (0.930,1.000) | 0.173 (0.085,0.259) |
|  |  | SSH | 0.102 (-0.081,0.268) | 1.000 (0.898,1.000) | 0.201 (0.095,0.302) |
| Substance abuse | cALC | NSSH | 0.023 (-0.316,0.339) | 0.999 (-1.000,1.000) | 0.189 (-0.053,0.336) |
|  |  | SSH | -0.082 (-0.531,0.325) | 0.991 (0.965,1.000) | 0.180 (-0.018,0.363) |
|  | cCANN | NSSH | 0.353 (0.353,0.680) | 0.852 (-1.000,1.000) | 0.164 (-0.090,0.590) |
|  |  | SSH | 0.466 (0.466,0.888) | 0.985 (-1.000,1.000) | 0.096 (-0.215,0.391) |
|  | cSMOK | NSSH | 0.253 (-0.010,0.538) | 0.981 (-1.000,1.000) | 0.276 (0.092,0.438) |
|  |  | SSH | 0.190 (-0.990,0.507) | 0.998 (-1.000,1.000) | 0.315 (0.117,0.572) |

# Table S9. Goodness of fit tests in trivariate analyses.

| Category | Measure | Model | -2 Log Likelihood | df | AIC | ∆ -2 Log Likelihood | ∆ df | p-value | q-value |
| --- | --- | --- | --- | --- | --- | --- | --- | --- | --- |
| Externalising problems | cSWAN | ACE model | 23036.39 | 19852 | -16667.6 | 4.32 | 3 | 0.229 | 0.610 |
|  |  | Constrained model | 23040.71 | 19855 | -16669.3 |  |  |  |  |
|  | pCONN | ACE model | 41725.73 | 25890 | -10054.3 | 2.07 | 3 | 0.559 | 0.789 |
|  |  | Constrained model | 41727.79 | 25893 | -10058.2 |  |  |  |  |
|  | pEMOL | ACE model | 40229.31 | 25890 | -11550.7 | 5.35 | 3 | 0.148 | 0.595 |
|  |  | Constrained model | 40234.66 | 25893 | -11551.3 |  |  |  |  |
|  | pHYPER | ACE model | 42996.61 | 25887 | -8777.39 | 2.22 | 3 | 0.528 | 0.789 |
|  |  | Constrained model | 42998.83 | 25890 | -8781.17 |  |  |  |  |
|  | pINAT | ACE model | 42909.11 | 25889 | -8868.89 | 8.08 | 3 | 0.044 | 0.356 |
|  |  | Constrained model | 42917.19 | 25892 | -8866.81 |  |  |  |  |
| Internalising problems | cANX | ACE model | 45759.79 | 25896 | -6032.21 | 0.61 | 3 | 0.893 | 0.991 |
|  |  | Constrained model | 45760.4 | 25899 | -6037.6 |  |  |  |  |
|  | cEAT | ACE model | 25519.81 | 19839 | -14158.2 | 3.28 | 3 | 0.350 | 0.617 |
|  |  | Constrained model | 25523.09 | 19842 | -14160.9 |  |  |  |  |
|  | cINSOM | ACE model | 36036.72 | 24115 | -12193.3 | 5.20 | 3 | 0.157 | 0.595 |
|  |  | Constrained model | 36041.93 | 24118 | -12194.1 |  |  |  |  |
|  | cMFQ | ACE model | 45289.22 | 25896 | -6502.78 | 2.16 | 3 | 0.541 | 0.789 |
|  |  | Constrained model | 45291.38 | 25899 | -6506.62 |  |  |  |  |
|  | pANX | ACE model | 28136.81 | 20905 | -13673.2 | 4.66 | 3 | 0.198 | 0.595 |
|  |  | Constrained model | 28141.48 | 20908 | -13674.5 |  |  |  |  |
|  | pMFQ | ACE model | 56337.82 | 25894 | 4549.82 | 9.85 | 3 | 0.020 | 0.356 |
|  |  | Constrained model | 56347.67 | 25897 | 4553.67 |  |  |  |  |
| Others | cAUT | ACE model | 46048.29 | 25889 | -5729.71 | 0.66 | 3 | 0.882 | 0.991 |
|  |  | Constrained model | 46048.95 | 25892 | -5735.05 |  |  |  |  |
|  | cSDQ | ACE model | 45951.91 | 25887 | -5822.09 | 3.35 | 3 | 0.341 | 0.617 |
|  |  | Constrained model | 45955.26 | 25890 | -5824.74 |  |  |  |  |
|  | pAUT | ACE model | 42388.26 | 25894 | -9399.74 | 0.32 | 3 | 0.957 | 0.991 |
|  |  | Constrained model | 42388.58 | 25897 | -9405.42 |  |  |  |  |
|  | pSDQ | ACE model | 45621.87 | 25899 | -6176.13 | 4.73 | 3 | 0.192 | 0.595 |
|  |  | Constrained model | 45626.61 | 25902 | -6177.39 |  |  |  |  |
| Psychotic-like experiences | cANHE | ACE model | 22611.77 | 25886 | -29160.2 | 8.94 | 3 | 0.030 | 0.356 |
|  |  | Constrained model | 22620.71 | 25889 | -29157.3 |  |  |  |  |
|  | cCAPS | ACE model | 42088.83 | 25898 | -9707.17 | 3.26 | 3 | 0.353 | 0.617 |
|  |  | Constrained model | 42092.09 | 25901 | -9709.91 |  |  |  |  |
|  | cGRAND | ACE model | 49025.66 | 25889 | -2752.34 | 0.11 | 3 | 0.991 | 0.991 |
|  |  | Constrained model | 49025.77 | 25892 | -2758.23 |  |  |  |  |
|  | cPRND | ACE model | 44502.34 | 25892 | -7281.66 | 5.44 | 3 | 0.142 | 0.595 |
|  |  | Constrained model | 44507.78 | 25895 | -7282.22 |  |  |  |  |
|  | cTEPS | ACE model | 71995.56 | 25897 | 20201.56 | 3.21 | 3 | 0.360 | 0.617 |
|  |  | Constrained model | 71998.78 | 25900 | 20198.78 |  |  |  |  |
|  | pSANS | ACE model | 48818.38 | 25888 | -2957.62 | 3.88 | 3 | 0.274 | 0.617 |
|  |  | Constrained model | 48822.26 | 25891 | -2959.74 |  |  |  |  |
| Substance abuse | cALC | ACE model | 21776.33 | 18573 | -15369.7 | 0.80 | 3 | 0.849 | 0.991 |
|  |  | Constrained model | 21777.14 | 18576 | -15374.9 |  |  |  |  |
|  | cCANN | ACE model | 17200.98 | 19226 | -21251 | 0.51 | 3 | 0.918 | 0.991 |
|  |  | Constrained model | 17201.49 | 19229 | -21256.5 |  |  |  |  |
|  | cSMOK | ACE model | 21512.15 | 19210 | -16907.9 | 1.78 | 3 | 0.620 | 0.827 |
|  |  | Constrained model | 21513.93 | 19213 | -16912.1 |  |  |  |  |
